# Enrichment of Melanoma Stem-Like Cells via Sphere Assays

**DOI:** 10.1007/978-1-0716-1205-7_14

**Published:** 2021

**Authors:** Nabanita Mukherjee, Karoline A. Lambert, David A. Norris, Yiqun G. Shellman

**Affiliations:** 1 Department of Dermatology, School of Medicine, University of Colorado Anschutz Medical Campus, Aurora, USA; 2 Dermatology Section, Department of Veterans Affairs Medical Center, Denver,USA; 3 Gates Center for Regenerative Medicine, University of Colorado Anschutz Medical Campus,Aurora,USA

**Keywords:** Melanoma stem cells, Cancer stem cell, Sphere assay, Cancer stemness, Melanoma-initiating cells, Cancer-initiating cells, Tumor spheres

## Abstract

Sphere assays are widely used in vitro techniques to enrich and evaluate the stem-like cell behavior of both normal and cancer cells. Utilizing three-dimensional in vitro sphere culture conditions provide a better representation of tumor growth in vivo than the more common monolayer cultures. We describe how to perform primary and secondary sphere assays, used for the enrichment and self-renewability studies of melanoma/melanocyte stem-like cells. Spheres are generated by growing melanoma cells at low density in nonadherent conditions with stem cell media. We provide protocols for preparing inexpensive and versatile polyHEMA-coated plates, setting up primary and secondary sphere assays in almost any tissue culture format and quantification methods using standard inverted microscopy. Our protocol is easily adaptable to laboratories with basic cell culture capabilities, without the need for expensive fluidic instruments.

## 1 Introduction

**Fig. 1:**
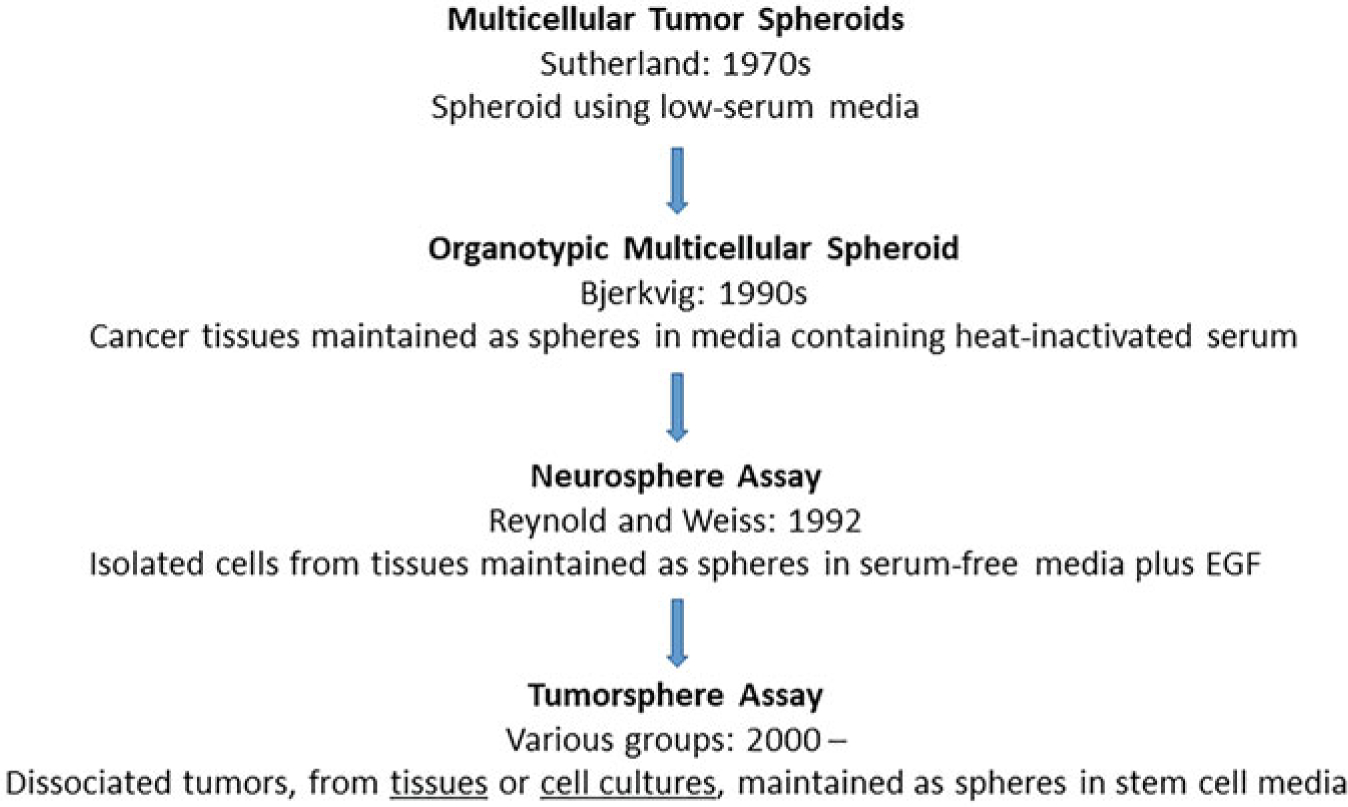
Major scientific breakthroughs leading to the current sphere assays

**Fig. 2:**
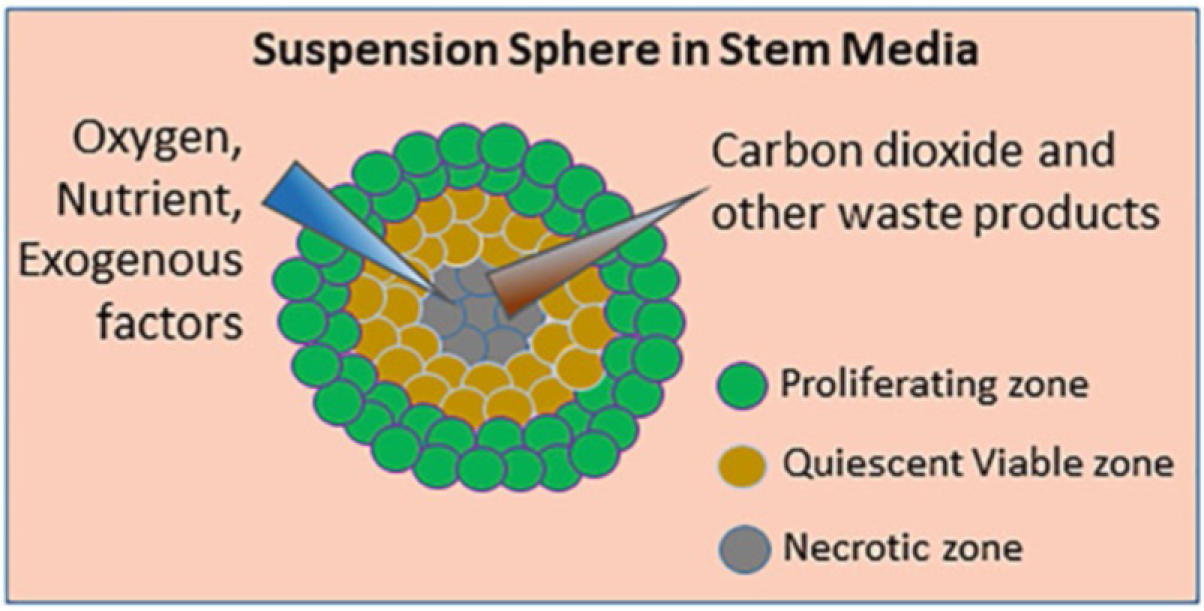
Schematic diagram of sphere 3D structure. Spheres have multiple layers of cells, each generating microenvironments to enable exchange of nutrient and waste products from the medium. (Figure adapted from [19])

The sphere formation assay is an in vitro functional assay for enriching adult stem cells and assessing their self-renewability potential [1–3]. Spheres have been widely used in stem and cancer research since the 1970s [2, 4–9] and are great functional assays complementary to assays based on cell surface stem-cell markers. A brief history of the development of this assay is provided in Fig. 1. In short, cells are grown in nonadherent, serum-free conditions to promote proliferation into three-dimensional spheres [1, 7, 9–17]. The media contains growth factors selective for stem-like cells, and anchorage-independent cultureware is used to push anoikis of non-stem cells, which are often dependent on adhesion or the extracellular matrix. Cells are seeded at low density, allowing for single cells to clonogenically proliferate into spheres. Each sphere forms a microenvironment within itself, with cell layers of different oxygenation, nutrition, and CO_2_ removal [18, 19] (Fig. 2). Additionally, primary spheres can be dissociated and re-seeded into the secondary sphere assay; only stem-like cells able to self-renew are able to re-form spheres. Therefore, the sphere, as a three-dimensional (3D) “tissue”, allows in vitro study of stem-like cells that closely resembles in vivo [2, 18, 20].

In cancer research, sphere assays are used to enrich the cancer stem cells (CSCs) of various solid cancers such as lung [21, 22], breast [23–26], ovarian [27–29], pancreatic [30–32], colon [33–35], liver [36], prostate [3, 37, 38], and melanoma [10, 11, 39–47, 53, 54]. Various experimental treatments can be applied to sphere assays as a means of analyzing the effects on the stem-like populations of cancers, including melanoma. For instance, in vitro multi-well plates of spheres can be used to test the effects—such as killing, resistance, or proliferation in response to inhibitors, growth factors, or genetic manipulations on the stem-like populations [40, 41, 44, 46–50]. Similar questions can be tested in vivo, by enriching for stem-like cells and implanting in a modified mouse xenograft model. For example, the tumor initiating frequency of melanoma-stem or initiating cells (MSCs/MICs) can be studied by enriching MSCs/MICs, followed by mouse implantation of very low cell numbers by serial dilution [44, 45, 51]. Similarly, the metastatic potential of CSC can be assessed with injection in the lateral tail vein of immunodeficient NOD *scid* gamma mice followed by the detection of the metastatic incidence in lungs [49].

**Fig. 3:**
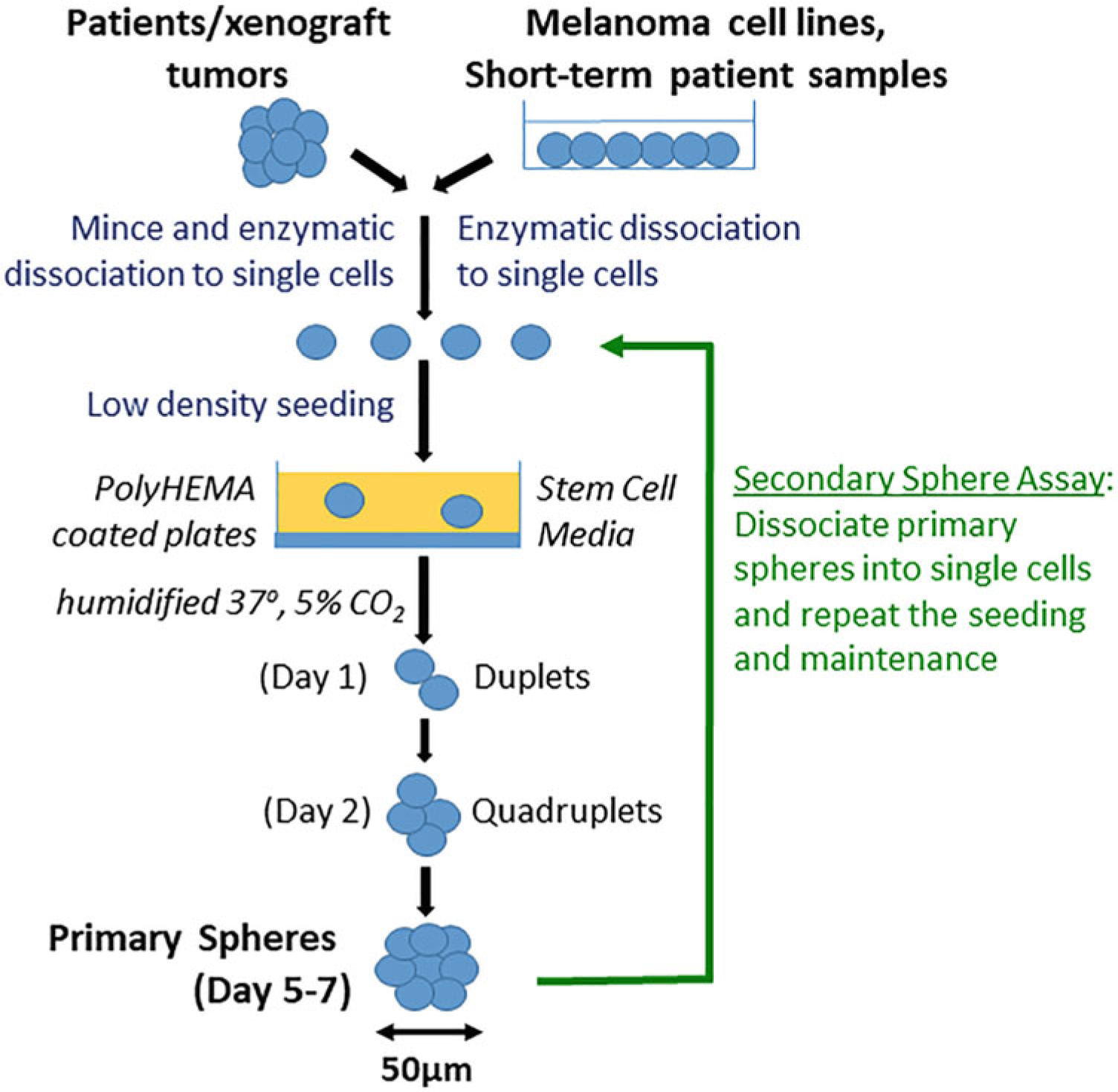
Schematic diagram of sphere assays

In this chapter, we describe the details of enriching MSC/MICs using sphere assays in suspension culture (Fig. 3). We have successfully used primary sphere assays to assess drug effects on melanoma stem-like cells [40, 44–46, 53, 54] and secondary sphere assays for self-renewal capacity [40, 44–46, 53, 54], and we have also implanted low number of cells from primary spheres in vivo to further assess tumor-initiating capacity of melanomas [44, 45]. Here, we explain the details of plate preparation, seeding, and quantification of sphere assays.

## 2 Materials

Whenever possible, use sterile technique and sterile reagents/supplies in a biosafety hood.

### 2.1 Standard Materials Necessary Throughout the Procedure

1. Micropipettes.
2. Sterile aerosol-resistant micropipette tips.
3. Pipet aid.
4. Serological pipettes.
5. Conical tubes (15 and 50 ml).
6. Benchtop centrifuge.

### 2.2 Preparing Non-adherent Plates for Sphere Assays

Sphere assays utilize anchorage-independent conditions. Culture can be done either in commercially available ultra-low attachment plates or in standard plates (tissue culture-treated or untreated) coated with polyHEMA. Commercially available plates are only available in 96-well, 24-well, and 6-well formats, whereas polyHEMA coating allows versatility in cultureware and is less expensive. Below are the materials needed to coat plates with polyHEMA:

1. Culture plates. We typically use 12-well, 6-well, 6-cm, or 10-cm plates.
2. 95% ETOH, diluted from 200 proof using sterile molecular grade water.
3. PolyHEMA (10×): 12% (w/v) of PolyHEMA in 95% ETOH, prepared in a sterile brown glass bottle. Zero the bottle on a scale in the biosafety hood. Add a small amount of polyHEMA powder, and weigh again to determine the weight of polyHEMA. Dilute to 12% (w/v) with 95% ETOH. Shake in 65 °C water bath for at least 4 h until visibly dissolved. Store at 4 °C (*see* Note 1).

### 2.3 Primary and Secondary Sphere Assays

The primary sphere assay enriches the MSCs/MICs from melanoma cells. We have successfully used this assay for commercially available melanoma cell lines, short-term cultures of patient samples, and tumors from mouse xenografts. The principle of this assay involves seeding the cells in nonadherent conditions using serum-free, stem cell medium to allow the formation of free-floating spheres. The size and number of spheres can be quantitated and compared between conditions, with or without treatments. The secondary sphere assay tests the self-renewal capacity of enriched MICs or is used for continuous maintenance of cells in sphere culture. We do the secondary sphere assay in 12-well non-adherent plates in triplicate for all conditions. These assays each take about 5–7 days (Fig. 3).

1. Melanoma cells (80–85% confluent).
2. Melanoma medium optimized for cells of interest (e.g., RPMI-1640 with 10% FBS).
3. Detachment solution optimized for cells of interest (e.g., Trypsin-EDTA (0.25%)).
4. Serum-free trypsin neutralization solution (e.g., defined trypsin inhibitor (DTI)).
5. DMEM/F12 medium.
6. B-27 Supplement (serum-free).
7. Penicillin–streptomycin solution (100×).
8. 20 ng/ml of human recombinant basic fibroblast growth factor (bFGF), prepared and stored according to manufacturer.
9. 20 ng/ml of human or mouse recombinant epidermal growth factor (EGF), prepared and stored according to manufacturer.
10. 4 μg/ml of heparin, prepared and stored according to manufacturer.
11. Stem cell medium, prepared fresh each time: DMEM/F12 with 2% B-27, 2× Pen/Strep, 40 pg/ml bFGF, 4 pg/ml EGF, and 480 pg/ml Heparin. To 47.9 ml of DMEM/F12, add 1 ml of B-27, 1 ml of Pen/Strep, 100 μl of bFGF, 10 μl of EGF, and 6 μl of heparin.
12. PolyHEMA-coated plates (prepared just prior to assay as described in Subheading 3.1).
13. Sterile PBS (1×): 137 mM NaCl, 2.7 mM KCl, 10 mM Na_2_HPO_4_, 1.8 mM KH_2_PO_4_.
14. 2 mM EDTA: Dilute 0.5 M EDTA stock with sterile PBS.
15. Inverted, optical light microscope with bright field and phase contrast options, with at least a 10× objective, ideally with a camera.
16. Automated cell counter or manual hand-held cell counter.
17. Trypan blue.
18. Sphere viability detection reagents: IncuCyte^®^ Caspase-3/7 Green Apoptosis Reagent, ethidium bromide, acridine orange, Annexin V, propidium iodide (optional).
19. IncuCyte^®^ S3 Live Cell Analysis System (Sartorius) (optional).
20. IncuCyte^®^ Cell-by-Cell Analysis Software Module (Sartorius Catalog #9600–0031) (optional).

## 3 Methods

### 3.1 Preparing PolyHEMA-Coated Plates

1. Right before coating plates (*see* Note 1), dilute 10× polyHEMA 1:10 in 95% ETOH. Pipette 1× polyHEMA onto plates as follows: 300 μl per well in 12-well plate; 0.5 ml per well in 6-well plate; 2.5 ml per 6 cm dish; 5 ml per 10 cm dish.
2. Place lids on plates and incubate in a dry 37 °C incubator (no water pan or cells) until dry. The small volumes in 12-well plates may dry overnight, but it may take 2–3 days to ensure all the ethanol has evaporated from larger plates.
3. Store coated plates at room temperature (plates can be stored for at least 6 months) and sterilize by UVC light right before seeding cells.

### 3.2 Seeding Primary Sphere Assay

The optimal seeding density is cell-specific (*see* Note 2) and ranges from 1000 cells/ml for fast-growing cell lines to 10,000 cells/ml for tumor samples. We recommend starting with a pilot study to determine the optimal density.

1. With the lids off, sterilize the polyHEMA-coated plates in biosafety hood UVC light for 20 min.
2. Freshly prepare the stem cell medium and warm up to 37°C in water bath. Volumes are standard for culture, e.g., 1 ml per well for 12-well plate, 2 ml per well for 6-well plate.
3. Detach adherent melanoma cells using method optimized for the particular cells, e.g., add trypsin–EDTA and place in the incubator for 5 min. For culturing spheres from human melanoma tumors and mouse tumors (*see* Notes 3 and 4).
4. Stop trypsin reaction with 2× volume of DTI.
5. Collect the cells in 15 ml tubes using about 8 ml of medium for a 10 cm dish of adherent cells. Spin conical tubes at 180 × *g* and discard the supernatant.
6. Resuspend the pellet in medium (volume depends on pellet size) by pipetting up and down multiple times to ensure there are no clumps.
7. Count cells and determine viability. We usually use a 1:10 dilution of cells while using an automated cell counter. Alternatively, manually count and determine viability with a trypan blue exclusion method, using a hemocytometer and a microscope. If cell clumping is evident during the cell count, pipette up and down and re-check for clumping. It is recommended that cells be at least 90% viable (*see* Note 5).
8. Plate viable cells at the recommended density (*see* Note 2) in stem cell medium (*see* Note 6).
9. Place the plates in a humidified incubator set at 37 °C with 5% CO_2_ (this seeding day represents day 0 of the assay).
10. Do not disturb the plates for 48 h after seeding as the spheres are especially fragile during the formation period and may dissociate due to mechanical movements. Handle plates very gently if they must be viewed or imaged for experimental purposes.

### 3.3 Maintenance of Primary Sphere Cultures

1. Feed cells with freshly prepared stem cell medium every 3–4 days, or when pH indicator turns yellow, by adding one-fourth the starting volume of medium to the plates, e.g., 250 μl of medium per well of a 12-well plate (*see* Note 7).
2. If testing the effects of drugs or other experimental treatments on spheres, *see* Note 8 regarding the timing of treatment.
3. View cultures with an inverted microscope to assess the formation of doublets and triplets 24–72 h after seeding [14] (*see* Note 9).

### 3.4 Seeding Secondary Sphere Assay

1. Transfer the primary spheres from plates into sterile 15 or 50 ml conical tubes using a wide bore pipette, such as 5- or 10-ml serological pipettes.
2. Wash the plates with an equal volume of sterile PBS and carefully collect any remaining spheres.
3. Centrifuge gently at low speed typical for cells, such as 180 × *g*, for 5 min at room temperature.
4. Aspirate off the media without disturbing the pellet.
5. Add 100–500 μl of 2 mM EDTA to the pellet and pipette up/down vigorously multiple times to dissociate the spheres into individual cells. The volume of EDTA depends on the pellet size.
6. Check the cells frequently under a light microscope to look for dissociation. Continue the process until at least 95% of spheres are dissociated.
7. Dilute the cell suspension with 1–10 ml of medium depending on the size of the pellet.
8. Count cells and determine viability using automated cell counter, or manually count and determine viability with a trypan blue exclusion method, using a hemocytometer and a microscope.
9. Plate the cells at the recommended density (*see* Notes 10–12) with stem cell medium.
10. Check the plates under the microscope right after seeding to ensure uniform seeding of single cells. To ensure uniform seeding, gently pipette up and down and tilt the plate in a circular motion.
11. Place the plate in a humidified incubator set at 37 °C with 5% CO_2_.
12. Maintain cultures and assess sphere formation according to the same timeline for Primary Sphere cultures described in Subheading 3.3 (*see* Fig. 3 and our publications [40, 44–46]).

### 3.5 Quantification of Spheres

1. Count the number of spheres in the entire well using an inverted microscope with a 10× objective, for a total magnification of 100× (*see* Note 13).
2. If analyzing differences in experimental treatments, report sphere data as a percentage by normalizing the sphere count of the treatment well respective to the sphere count of the control well [40, 44–46]. Alternatively, report the sphere efficiency as the number of spheres formed divided by the total number of cells seeded [14].
3. Capture images of spheres with camera fitted to microscope if desirable.

### 3.6 Evaluation of Sphere Viability

It is often necessary to analyze the viability or apoptosis of the cells, especially in treatment experiments. Below are some easy and useful ways to evaluate viability of spheres.

1. Perform live-cell imaging with the IncuCyte^®^ S3 Live Cell Analysis System or a related imaging system in order to visualize the formation and development of spheres over time—the addition of IncuCyte^®^ Caspase-3/7 Green Apoptosis Reagent allows for the analysis of live/dead populations over time (*see* Note 14).
2. Perform dual ethidium bromide and acridine orange (EB/AO) staining, which is a low-cost alternative to live cell imaging that also distinguishes apoptotic cells from necrotic cells [52] (*see* Note 15).
3. Dissociate spheres into single cells and analyze by conventional assays, such as flow cytometry with Annexin-V/propidium iodide.

## 4 Notes

**Fig. 4:**
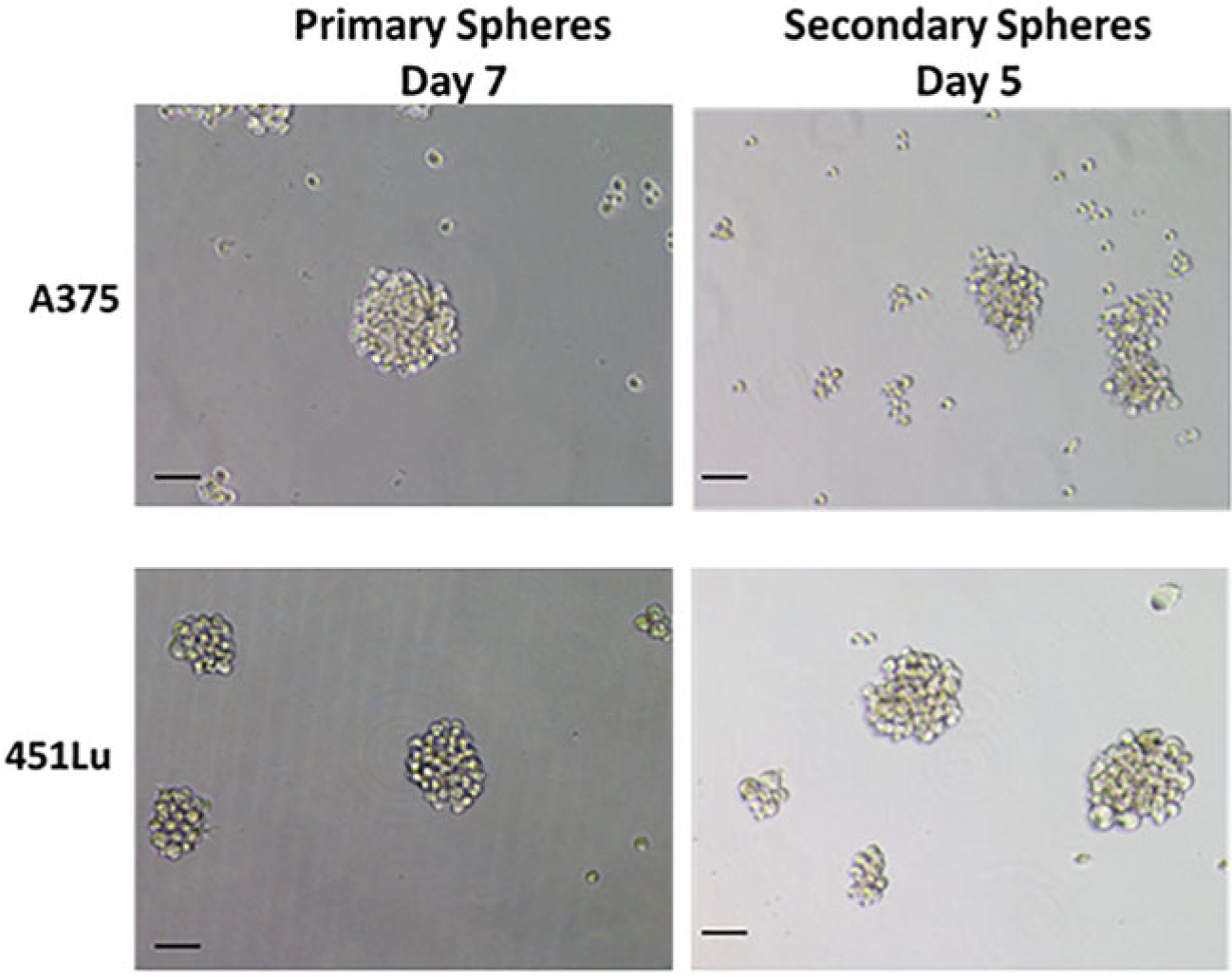
Examples of primary and secondary spheres from melanoma cells. The ideal spheres are round and tight. The secondary spheres grow much faster and reach similar size in a shorter time. Scale bar = 100 μm

**Fig. 5:**
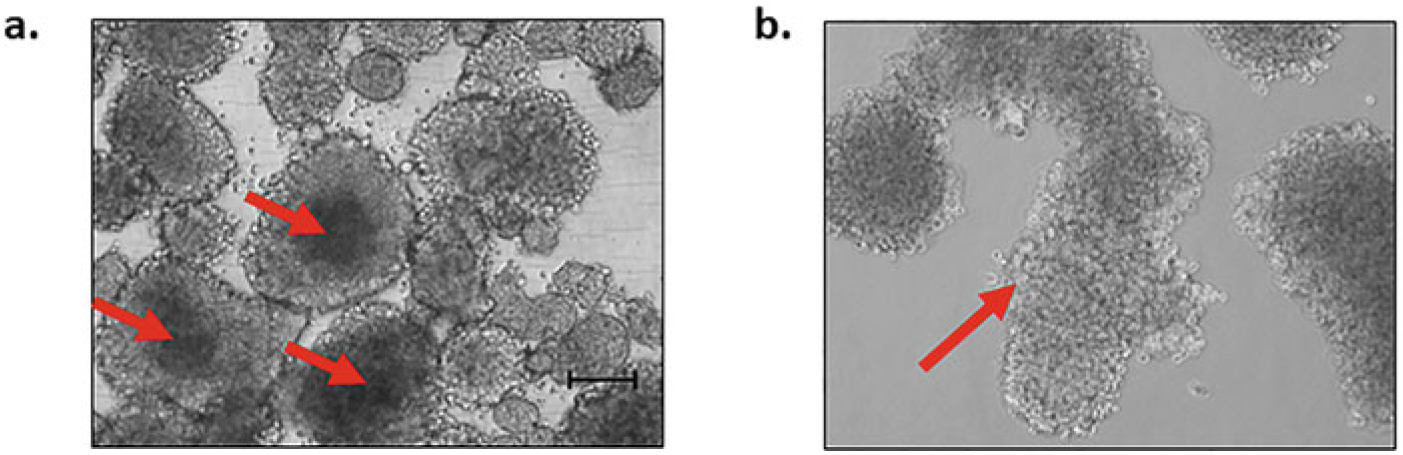
Examples of overgrown, difficult to count, spheres. (**a**) Overgrown spheres with necrotic areas. Spheres allowed to proliferate larger than 200 μm diameter often show necrotic centers (red arrow). (**b**) Spheres which are stuck together (red arrow) due to over proliferation. Scale bar = 100 μm

1. If you are using a 10× stock of polyHEMA stored at 4 °C, warm up by shaking at least 1 h in 65°C water bath.
2. For sphere assays, the cells need to be seeded at a lower density than typical for regular monolayer culture. The majority of commercially available melanoma cells form spheres when seeded at a density of 1000–5000 viable cells/ml. For instance, the fast-growing melanoma line A375 can be seeded at 1000 viable cells/ml, while the slower growing line HT-144 can be seeded at a density of 5000 viable cells/ml. For patient-derived melanoma cells, the density may need to be higher (5000–10,000 viable cells/ml) depending on the growth rate of cells.
3. For culturing spheres from human melanoma tumors, the tumors need to be minced thoroughly and may need to be enzymatically dissociated until a homogeneous mixture is obtained. The cells often need to be seeded at a higher density of 5000 cells per ml of stem cell medium [12].
4. For culturing cells from mouse xenograft tumors of nude mice, the tumor tissue needs to be minced and dissociated as described in Note 3. After dissociation and cell count, it is important to separate out mouse cells from human cells by magnetic activated cell sorting (MACS), which we achieve with the commercially available Mouse Cell Depletion Kit (Miltenyi Biotec).
5. If cell clumping is evident during the cell count, pipette up and down and re-check for clumping. It is recommended that cells be at least 90% viable.
6. We recommend diluting cells in a 50 ml falcon tube, followed by gentle shaking or pipetting up and down to ensure mixing. Add the volume of cell of suspension to well or dish. It is helpful to tilt the plates and tap at the bottom gently to ensure spreading of cells.
7. Unlike standard procedures for regular 2D cell culture, do not attempt to exchange medium, as the mechanical stress of pipetting might dissociate the spheres. Instead, add extra medium gently from the side of the well or dish.
8. For testing the efficacy of certain drugs in MSC/MIC population, the drug should be added by day 5. This will enable better visualization of drug effect in vehicle and test wells without over-confluence of the vehicle wells. At least three replicates for each study/experimental condition are recommended.
9. Small compact spheroid masses of cells will be visible around day 4. By days 5–7, well-developed spheres of 50 μm or more in diameter should be visible (Fig. 4). This is the ideal time to count spheres. Once the spheres are of diameter 200 μm or larger, the cells will undergo apoptosis and appear dark in the center (Fig. 5). At this point, it is better to passage the cells into the secondary (or tertiary) sphere assay.
10. We recommend seeding the secondary spheres at the same density as primary spheres. However, for some cells, the secondary spheres grow faster (Fig. 4).
11. Sphere assays can be conducted in any sized culture plates or flasks. However, 6-well or 12-well plates are recommended for quantification, and larger 10-cm dishes are recommended for protein lysates.
12. For secondary sphere assays, we find it ideal to do triplicate wells in 12-well plates for each condition. To ensure enough viable cells for the secondary sphere assay, it is important to seed cells for the primary spheres in larger plates. For instance, we typically set up primary sphere assays in 6-cm or 10-cm plates, which ensures enough cells for triplicate conditions in the secondary sphere assay, as well as for lysate collection. Also, if looking at the effect of drugs on primary spheres, expect much death, and seed the primary experiment accordingly.
13. Sharpies can be used to divide the plate into multiple areas for counting. For each well of 12-well plate, we divide the entire well into ten fractions for counting. The optimum time to count the spheres is typically between days 4 and 7. Spheres are easier to count if seeded in 6-well or 12-well plates, as it is easier to keep track of your place in the plate, thereby avoiding double counting. Pay attention to the sphere quality and relative size, and take images of the representative spheres if desired and able. It is important to distinguish spheres from loose cell aggregates. If there is doubt regarding a sphere versus an aggregate, it is useful to shake the plate very gently. A loose aggregate will dismantle, while a sphere will not. For faster growing cell lines, it is best to do the quantifications when the spheres are 100 μm in diameter. If the spheres are allowed to grow for longer, they over-proliferate and stick to one another.
14. Images are collected using phase contrast microscopy and a standard green fluorescence channel at time intervals, for multiple days. The same process can also be used to visualize the effects of compounds in disrupting the already formed spheres. The accompanying Incucyte^®^ Cell-by-Cell Analysis Software Module is intuitive and automates sphere counts and viability analysis.
15. EB/AO is simply added to the culture media and immediately visualized under an inverted microscope with a standard green fluorescence channel. Live or necrotic cells fluoresce green or red, respectively, and apoptotic cells show green blebbing of the membrane. These methods have been described and quantitatively used in melanoma and other studies [40, 44, 45]. EB/AO are mutagenic compounds; care must be used when handling, and waste must be disposed of according to local regulations.

## Abbreviations

CICs: Cancer-initiating cells
CSCs: Cancer stem cells
MICs: Melanoma-initiating cells
MSCs: Melanoma stem cells

## References

[1] Pastrana E, Silva-Vargas V, Doetsch F (2011) Eyes wide open: a critical review of sphere-formation as an assay for stem cells. Cell Stem Cell 8(5):486–498. 10.1016/j.stem.2011.04.007 21549325 PMC3633588

[2] Weiswald L-B, Bellet D, Dangles-Marie V (2015) Spherical cancer models in tumor biology. Neoplasia 17(1):1–15. 10.1016/j.neo.2014.12.004 25622895 PMC4309685

[3] Bahmad HF, Cheaito K, Chalhoub RM, Hadadeh O, Monzer A, Ballout F, El-Hajj A, Mukherji D, Liu Y-N, Daoud G, Abou-Kheir W (2018) Sphere-formation assay: three-dimensional in vitro culturing of prostate cancer stem/progenitor sphere-forming cells. Front Oncol 8(347). 10.3389/fonc.2018.00347 PMC6121836 30211124

[4] Inch WR, McCredie JA, Sutherland RM (1970) Growth of nodular carcinomas in rodents compared with multi-cell spheroids in tissue culture. Growth 34(3):271–282 5471822

[5] Sutherland RM, Inch WR, McCredie JA, Kruuv J (1970) A multi-component radiation survival curve using an in vitro tumour model. Int J Radiat Biol Relat Stud Phys Chem Med 18(5):491–495. 10.1080/09553007014551401 5316564

[6] Bjerkvig R, Tonnesen A, Laerum OD, Backlund EO (1990) Multicellular tumor spheroids from human gliomas maintained in organ culture. J Neurosurg 72(3):463–475. 10.3171/jns.1990.72.3.0463 2406382

[7] Reynolds BA, Weiss S (1992) Generation of neurons and astrocytes from isolated cells of the adult mammalian central nervous system. Science 255(5052):1707. 10.1126/science.1553558 1553558

[8] Kaaijk P, Troost D, Das PK, Leenstra S, Bosch DA (1995) Long-term culture of organotypic multicellular glioma spheroids: a good culture model for studying gliomas. Neuropathol Appl Neurobiol 21(5):386–391. 10.1111/j.1365-2990.1995.tb01075.x 8632833

[9] Dou J, Pan M, Wen P, Li Y, Tang Q, Chu L, Zhao F, Jiang C, Hu W, Hu K, Ning G (2008) Isolation and identification of cancer stem like cells from murine melanoma cell lines. Cell Mol Immunol 4(6):467–472 18163959

[10] Schatton T, Frank M (2010) The in vitro spheroid melanoma cell culture assay: cues on tumor initiation? J Invest Dermatol 130:1769–1771. 10.1038/jid.2010.135 20548315 PMC2902551

[11] Girouard SD, Murphy GF (2011) Melanoma stem cells: not rare, but well done. Lab Invest 91(5):647–664. 10.1038/labinvest.2011.50 21445060

[12] Stecca B, Santini R, Pandolfi S, Penachioni JY (2013) Culture and isolation of melanoma-initiating cells. Curr Protoc Stem Cell Biol Chapter 3:Unit 3.6. 10.1002/9780470151808.sc0306s24 23404674

[13] Nassar D, Blanpain C (2016) Cancer stem cells: basic concepts and therapeutic implications. Annu Rev Pathol 11(1):47–76. 10.1146/annurev-pathol-012615-044438 27193450

[14] Tuccitto A, Beretta V, Rini F, Castelli C, Perego M (2017) Melanoma stem cell sphere formation assay. Bio-protocol 7. 10.21769/BioProtoc.2233 PMC8410273 34541232

[15] Phi LTH, Sari IN, Yang YG, Lee SH, Jun N, Kim KS, Lee YK, Kwon HY (2018) Cancer stem cells (CSCs) in drug resistance and their therapeutic implications in cancer treatment. Stem Cells Int 2018:5416923. 10.1155/2018/5416923 29681949 PMC5850899

[16] Capp JP (2019) Cancer stem cells: from historical roots to a new perspective. J Oncol 2019:5189232. 10.1155/2019/5189232 31308849 PMC6594320

[17] Atashzar MR, Baharlou R, Karami J, Abdollahi H, Rezaei R, Pourramezan F, Zoljalali Moghaddam SH (2020) Cancer stem cells: a review from origin to therapeutic implications. J Cell Physiol 235(2):790–803. 10.1002/jcp.29044 31286518

[18] Ham SL, Joshi R, Thakuri PS, Tavana H (2016) Liquid-based three-dimensional tumor models for cancer research and drug discovery. Exp Biol Med (Maywood) 241(9):939–954. 10.1177/1535370216643772 27072562 PMC4950350

[19] Chaicharoenaudomrung N, Kunhorm P, Noisa P (2019) Three-dimensional cell culture systems as an in vitro platform for cancer and stem cell modeling. World J Stem Cells 11 (12):1065–1083. 10.4252/wjsc.v11.i12.1065 31875869 PMC6904866

[20] Deleyrolle LP, Ericksson G, Morrison BJ, Lopez JA, Burrage K, Burrage P, Vescovi A, Rietze RL, Reynolds BA (2011) Determination of somatic and cancer stem cell self-renewing symmetric division rate using sphere assays. PLoS One 6(1):e15844–e15844. 10.1371/journal.pone.0015844 21246056 PMC3016423

[21] Pan J, Zhang Q, Wang Y, You M (2010) 26S proteasome activity is down-regulated in lung cancer stem-like cells propagated in vitro. PLoS One 5(10):e13298–e13298. 10.1371/journal.pone.0013298 20949018 PMC2952619

[22] Herreros-Pomares A, de-Maya-Girones JD, Calabuig-Fariñas S, Lucas R, Martínez A, Pardo-Sánchez JM, Alonso S, Blasco A, Guijarro R, Martorell M, Escorihuela E, Chiara MD, Duréndez E, Gandía C, Forteza J, Sirera R, Jantus-Lewintre E, Farràs R, Camps C (2019) Lung tumor spheres reveal cancer stem cell-like properties and a score with prognostic impact in resected non-small-cell lung cancer. Cell Death Dis 10(9):660. 10.1038/s41419-019-1898-1 31506430 PMC6737160

[23] Dontu G, Abdallah WM, Foley JM, Jackson KW, Clarke MF, Kawamura MJ, Wicha MS (2003) In vitro propagation and transcriptional profiling of human mammary stem/progenitor cells. Genes Dev 17(10):1253–1270. 10.1101/gad.1061803 12756227 PMC196056

[24] Duru N, Fan M, Candas D, Menaa C, Liu H-C, Nantajit D, Wen Y, Xiao K, Eldridge A, Chromy BA, Li S, Spitz DR, Lam KS, Wicha MS, Li JJ (2012) HER2-associated radioresistance of breast cancer stem cells isolated from HER2-negative breast cancer cells. Clin Cancer Res 18(24):6634–6647. 10.1158/1078-0432.CCR-12-1436 23091114 PMC3593096

[25] Tsuyada A, Chow A, Wu J, Somlo G, Chu P, Loera S, Luu T, Li AX, Wu X, Ye W, Chen S, Zhou W, Yu Y, Wang Y-Z, Ren X, Li H, Scherle P, Kuroki Y, Wang SE (2012) CCL2 mediates cross-talk between cancer cells and stromal fibroblasts that regulates breast cancer stem cells. Cancer Res 72(11):2768–2779. 10.1158/0008-5472.CAN-11-3567 22472119 PMC3367125

[26] Lombardo Y, de Giorgio A, Coombes CR, Stebbing J, Castellano L (2015) Mammosphere formation assay from human breast cancer tissues and cell lines. J Vis Exp 97:52671. 10.3791/52671 PMC4401367 25867607

[27] He Q, Luo X, Wang K, Zhou Q, Ao H, Yang Y, Sx L, Li Y, Zhu H, Duan T (2014) Isolation and characterization of cancer stem cells from high-grade serous ovarian carcinomas. Cell Physiol Biochem 33(1):173–184. 10.1159/000356660 24504111

[28] Wang H, Paczulla A, Lengerke C (2015) Evaluation of stem cell properties in human ovarian carcinoma cells using multi and single cell-based spheres assays. J Vis Exp 95: e52259–e52259. 10.3791/52259 PMC4354514 25590994

[29] Dong P, Xiong Y, Watari H, Hanley SJB, Konno Y, Ihira K, Yamada T, Kudo M, Yue J, Sakuragi N (2016) MiR-137 and miR-34a directly target Snail and inhibit EMT, invasion and sphere-forming ability of ovarian cancer cells. J Exp Clin Cancer Res 35(1):132. 10.1186/s13046-016-0415-y 27596137 PMC5011787

[30] Gou S, Liu T, Wang C, Yin T, Li K, Yang M, Zhou J (2007) Establishment of clonal colonyforming assay for propagation of pancreatic cancer cells with stem cell properties. Pancreas 34(4):429–435. 10.1097/MPA.0b013e318033f9f4 17446842

[31] Bao B, Azmi AS, Aboukameel A, Ahmad A, Bolling-Fischer A, Sethi S, Ali S, Li Y, Kong D, Banerjee S, Back J, Sarkar FH (2014) Pancreatic cancer stem-like cells display aggressive behavior mediated via activation of FoxQ1. J Biol Chem 289(21):14520–14533. 10.1074/jbc.M113.532887 24719318 PMC4031510

[32] Xu Z, Jia Y, Huang X, Feng N, Li Y (2018) Rapid induction of pancreatic cancer cells to cancer stem cells via heterochromatin modulation. Cell Cycle 17(12):1487–1495. 10.1080/15384101.2018.1489180 30045656 PMC6132961

[33] Qureshi-Baig K, Ullmann P, Rodriguez F, Frasquilho S, Nazarov PV, Haan S, Letellier E (2016) What do we learn from spheroid culture systems? Insights from tumorspheres derived from primary colon cancer tissue. PLoS One 11(1):e0146052. 10.1371/journal.pone.0146052 26745821 PMC4706382

[34] Olejniczak A, Szarynska M, Kmiec Z (2018) In vitro characterization of spheres derived from colorectal cancer cell lines. Int J Oncol 52 (2):599–612. 10.3892/ijo.2017.4206 29207035

[35] Zhao H, Yan C, Hu Y, Mu L, Huang K, Li Q, Li X, Tao D, Qin J (2019) Sphereforming assay vs. organoid culture: determining longterm stemness and the chemoresistant capacity of primary colorectal cancer cells. Int J Oncol 54(3):893–904. 10.3892/ijo.2019.4683 30664193 PMC6365025

[36] Ma X-L, Sun Y-F, Wang B-L, Shen M-N, Zhou Y, Chen J-W, Hu B, Gong Z-J, Zhang X, Cao Y, Pan B-s, Zhou J, Fan J, Guo W, Yang X-R (2019) Sphere-forming culture enriches liver cancer stem cells and reveals Stearoyl-CoA desaturase 1 as a potential therapeutic target. BMC Cancer 19(1):760. 10.1186/s12885-019-5963-z 31370822 PMC6676608

[37] Mulholland DJ, Xin L, Morim A, Lawson D, Witte O, Wu H (2009) Lin-Sca-1+CD49fhigh stem/progenitors are tumor-initiating cells in the Pten-null prostate cancer model. Cancer Res 69(22):8555–8562. 10.1158/0008-5472.CAN-08-4673 19887604 PMC2783355

[38] Lukacs RU, Memarzadeh S, Wu H, Witte ON (2010) Bmi-1 is a crucial regulator of prostate stem cell self-renewal and malignant transformation. Cell Stem Cell 7(6):682–693. 10.1016/j.stem.2010.11.013 21112563 PMC3019762

[39] Santini R, Vinci MC, Pandolfi S, Penachioni JY, Montagnani V, Olivito B, Gattai R, Pimpinelli N, Gerlini G, Borgognoni L, Stecca B (2012) Hedgehog-GLI signaling drives self-renewal and tumorigenicity of human melanoma-initiating cells. Stem Cells 30 (9):1808–1818. 10.1002/stem.1160 22730244

[40] Mukherjee N, Reuland SN, Lu Y, Luo Y, Lambert K, Fujita M, Robinson WA, Robinson SE, Norris DA, Shellman YG (2015) Combining a BCL2 inhibitor with the retinoid derivative fenretinide targets melanoma cells including melanoma initiating cells. J Invest Dermatol 135(3):842–850. 10.1038/jid.2014.464 25350317 PMC4323853

[41] Marzagalli M, Moretti RM, Messi E, Marelli MM, Fontana F, Anastasia A, Bani MR, Beretta G, Limonta P (2018) Targeting melanoma stem cells with the vitamin E derivative δ-tocotrienol. Sci Rep 8(1):587. 10.1038/s41598-017-19057-4 29330434 PMC5766483

[42] Mukherjee N, Schwan JV, Fujita M, Norris DA, Shellman YG (2015) Alternative treatments for melanoma: targeting BCL-2 family members to de-bulk and kill cancer stem cells. J Invest Dermatol 135(9):2155–2161. 10.1038/jid.2015.145 25947358 PMC4537369

[43] Nguyen N, Couts KL, Luo Y, Fujita M (2015) Understanding melanoma stem cells. Melanoma Manag 2(2):179–188. 10.2217/mmt.15.4 26594315 PMC4649940

[44] Mukherjee N, Almeida A, Partyka KA, Lu Y, Schwan JV, Lambert K, Rogers M, Robinson WA, Robinson SE, Applegate AJ, Amato CM, Luo Y, Fujita M, Norris DA, Shellman YG (2016) Combining a GSI and BCL-2 inhibitor to overcome melanoma’s resistance to current treatments. Oncotarget 7(51):84594–84607. 10.18632/oncotarget.13141 27829238 PMC5356684

[45] Mukherjee N, Lu Y, Almeida A, Lambert K, Shiau CW, Su JC, Luo Y, Fujita M, Robinson WA, Robinson SE, Norris DA, Shellman YG (2017) Use of a MCL-1 inhibitor alone to de-bulk melanoma and in combination to kill melanoma initiating cells. Oncotarget 8 (29):46801–46817. 10.18632/oncotarget.8695 27086916 PMC5564524

[46] Mukherjee N, Strosnider A, Vagher B, Lambert KA, Slaven S, Robinson WA, Amato CM, Couts KL, Bemis JGT, Turner JA, Norris DA, Shellman YG (2018) BH3 mimetics induce apoptosis independent of DRP-1 in melanoma. Cell Death Dis 9(9):907. 10.1038/s41419-018-0932-z 30185782 PMC6125485

[47] Pietrobono S, Morandi A, Gagliardi S, Gerlini G, Borgognoni L, Chiarugi P, Arbiser JL, Stecca B (2016) Down-regulation of SOX2 underlies the inhibitory effects of the triphenylmethane gentian violet on melanoma cell self-renewal and survival. J Invest Dermatol 136 (10):2059–2069. 10.1016/j.jid.2016.06.610 27373978

[48] Yang F, Wei J, Zhang S, Zhang X (2017) Shrimp miR-S8 suppresses the stemness of human melanoma stem-like cells by targeting the transcription factor YB-1. Cancer Res 77 (20):5543–5553. 10.1158/0008-5472.Can-17-1375 28855207

[49] Wang Y, Leonard M, Snyder D, Fisher M, Eckert R, Kaetzel D (2019) NME1 drives expansion of melanoma cells with enhanced tumor growth and metastatic properties. Mol Cancer Res 17(8):1665–1674. 10.1158/1541-7786.MCR-18-0019 31123173 PMC6677611

[50] Tuccitto A, Tazzari M, Beretta V, Rini F, Miranda C, Greco A, Santinami M, Patuzzo R, Vergani B, Villa A, Manenti G, Cleris L, Giardiello D, Alison M, Rivoltini L, Castelli C, Perego M (2016) Immunomodulatory factors control the fate of melanoma tumor initiating cells. Stem Cells 34(10):2449–2460. 10.1002/stem.2413 27301067

[51] Roesch A, Fukunaga-Kalabis M, Schmidt EC, Zabierowski SE, Brafford PA, Vultur A, Basu D, Gimotty P, Vogt T, Herlyn M (2010) A temporarily distinct subpopulation of slowcycling melanoma cells is required for continuous tumor growth. Cell 141(4):583–594. 10.1016/j.cell.2010.04.020 20478252 PMC2882693

[52] Ribble D, Goldstein NB, Norris DA, Shellman YG (2005) A simple technique for quantifying apoptosis in 96-well plates. BMC Biotechnol 5:12. 10.1186/1472-6750-5-12 15885144 PMC1142306

[53] Mukherjee N, Amato CM, Skees J, Todd KJ, Lambert KA, Robinson WA, Van Gulick R, Weight RM, Dart CR, Tobin RP, McCarter MD, Fujita M, Norris DA, Shellman YG (2020) Simultaneously Inhibiting BCL2 and MCL1 Is a Therapeutic Option for Patients with Advanced Melanoma. Cancers (Basel) 12 (8). 10.3390/cancers12082182 PMC7464298 32764384

[54] Mukherjee N, Skees J, Todd KJ, West DA, Lambert KA, Robinson WA, Amato CM, Couts KL, Van Gulick R, MacBeth M, Nassar K, Tan A-C, Zhai Z, Fujita M, Bagby SM, Dart CR, Lambert JR, Norris DA, Shellman YG (2020) MCL1 inhibitors S63845/MIK665 plus Navitoclax synergistically kill difficult-to-treat melanoma cells. Cell death & disease 11 (6):443. 10.1038/s41419-020-2646-2 32513939 PMC7280535

